# Development, Validity, and Reliability of the Perceived Telemedicine Importance, Disadvantages, and Barriers (PTIDB) Questionnaire for Egyptian Healthcare Professionals

**DOI:** 10.3390/ijerph191912678

**Published:** 2022-10-04

**Authors:** Naglaa Youssef, Ramy Mohamed Ghazy, Reem Ezzat Mahdy, Mohammad Abdalgabar, Omar Elshaarawy, Mohamed Alboraie

**Affiliations:** 1 Department of Medical-Surgical Nursing, College of Nursing, Princess Nourah Bint Abdulrahman University, P.O. Box 84428, Riyadh 11671, Saudi Arabia; 2 Tropical Health Department, High Institute of Public Health, Alexandria University, Alexandria 21561, Egypt; 3 Department of Internal Medicine, Assiut University, Assiut 71515, Egypt; 4 Department of Gastroenterology, Police Authority Hospital, Giza 0000, Egypt; 5 Hepatology and Gastroenterology Department, National Liver Institute, Menoufia University, Menofia 32511, Egypt; 6 Department of Internal Medicine, Al-Azhar University, Cairo 11884, Egypt

**Keywords:** telemedicine, validity, reliability, questionnaire, Arabic

## Abstract

Background: This study aimed to develop and investigate the psychometric properties of the Perceived Telemedicine Importance, Disadvantages, and Barriers (PTIDB) questionnaire for healthcare professionals (HCPs) in Egypt. This study was conducted in three phases: (1) development of the questionnaire, (2) preliminary testing of the questionnaire, and (3) investigation of its validity and reliability using a large survey. Methods: A cross-sectional survey was conducted over two months. A convenience sample of 691 HCPs and clerks from 22 governorates accessed the online survey. The construct validity was assessed using exploratory factor analysis (EFA), confirmatory factor analysis (CFA), and internal reliability. Results: The initial Eigenvalues showed that all 19 items of the questionnaire explained 56.0% of the variance in three factors. For Factor 1 (importance), eight items were loaded on one factor, with factor loading ranging from 0.61 to 0.78. For Factor 2 (disadvantages), seven items were loaded on one factor with factor loading ranging from 0.60 to 0.79. For Factor 3 (barriers), four items were loaded on one factor, with factor loading ranging from 0.60 to 0.86. The CFA showed that All loadings ranged from 0.4 to 1.0, with CFI = 0.93 and RMSEA = 0.061. All the factors had satisfactory reliability; 0.87 for ‘‘Importance’’, 0.82 for ‘‘Disadvantages’’, and 0.79 for ‘‘Barriers’’. Conclusion: The PTIDB questionnaire has an acceptable level of validity and internal consistency, at a readability level of 12th grade. The retest reliability, however, still needs to be tested.

## 1. Introduction

Coronavirus disease 2019 (COVID-19) is an infectious disease that occurred in 2019 as a result of a contagious virus that was known as ‘‘severe acute respiratory syndrome coronavirus 2 (SARS-CoV-2)’’ [1]. In December 2019, the first cases of COVID-19 were reported in Wuhan, China [1]; afterwards, the virus quickly spread around the world.

On 11 March 2020, the opening remarks of the Director-General of the World Health Organization (WHO) declared COVID-19 to be a global pandemic [2]. By September 21, 2022, 610,393,563 confirmed cases of COVID-19 had been reported to the WHO, including 6,508,521 mortality cases, with a variation in the pattern of infection and death rates across countries [3]. In Egypt, the first case of COVID-19 was reported on 14 February 2020; by the first of August 2020, the number of cases had increased to over 95,000 [4,5]. Since then, the COVID-19 pandemic has spread to several governorates in Egypt, resulting in 515,361 confirmed cases and 24,797 deaths due to COVID-19, as reported by the WHO on September 21, 2022 [6].

Since the spread of COVID-19 began in Egypt, the Egyptian government has taken early preventive measures to minimize human and economic losses due to the pandemic [4]. On March 10, 2020, after the number of confirmed COVID-19 cases increased above 100, Egypt took action to lock-down shops as well as places of social activity and entertainment. Educational institutes were shifted online, a partial curfew was enforced, and transportation was suspended [7,8]. Several quarantine hospitals were allocated across Egypt, and infection control measures were implemented gradually [8]. Healthcare services were also affected since doctors and nurses were busy caring for COVID-19 cases, and hospitals were occupied with confirmed cases owing to low resources [7,9]. Additionally, the extension of the partial lockdown period forced many physicians to close their clinics, and hospitals provided services only for real emergencies, as the scarcity of supplies limited the access to healthcare facilities for the general population [7].

To mitigate the impact of COVID-19 on population health, many countries, including Egypt, have introduced telemedicine as a way for health professionals to offer medical advice and follow-up with their patients [10,11]. Telemedicine has been acknowledged as a supportive tool to traditional medicine because it can facilitate the delivery of healthcare to service beneficiaries when social distancing is compulsory [10]. For that reason, it has been used extensively during the COVID-19 pandemic by healthcare professionals (HCPs) as it facilitates connecting, transmitting, and exchanging patients’ health information; conducting specialist consultations; and getting advice from other specialists [9,11]. Moreover, it could enable HCPs to assess and follow up with their patients remotely using computed tomography devices, and exchange patients’ health information and medical records synchronously [12]. During the COVID-19 pandemic, telemedicine has improved access to high-quality healthcare and decreased infection risk in low- and middle-income countries (LMICs) [9]; allowing the establishment of telemedicine and enhancing its acceptance by the HCPs and the general population.

A recent scoping review found that over the first six months of COVID-19, the considerable emerging literature on telemedicine has been published mostly from high-income countries [11]. Thus, understanding the barriers to implementing telemedicine in these countries requires further attention.

In 1997, the WHO provided a broad definition of telemedicine: “the delivery of healthcare services, where distance is a critical factor, by all HCPs using information and communication technologies for the exchange of valid information for the diagnosis, treatment, and prevention of disease and injuries, research and evaluation, and for the continuing education of HCPs, all in the interests of advancing the health of individuals and their communities” [13] (p. 11). Since then, telemedicine strategies have been developed and recommended as inexpensive methods that could support health services and related services, such as screening, health education, and healthcare [14]. Telemedicine has been reported as an essential way to streamline outpatient visits, which can reduce costs, with significant benefits for the public health service budget in Italy [10], which can offer valuable support to the healthcare providers’ activities by easing their work. Telemedicine facilitates remote communication, exchange of experience, and transfer of information or medical records, images, and laboratory results using electronic devices such as mobiles, laptops, or computers [9].

Transformation of information and communication in telemedicine is usually conducted by using synchronizes and desynchronizes depending on the timing of the information that is transmitted and the communication between HCPs and their patients [15]. Synchronic refers to the interaction between HCPs and patients via voice or video calls, or even instant text messages, and has been the most common method of communication. In contrast, desynchronized communication is most often conducted by meeting at different times, for example, by sending messages via email or broadcasting [9,15].

Despite evidence supporting the implementation of telemedicine in developed countries, as it can provide high-quality healthcare at an affordable cost [16,17,18], numerous infrastructure, socioeconomic, and regulatory barriers affect its use [9,18], especially in LMICs [18]. Telemedicine is possibly useful in the management of patients who have chronic conditions and who require continuous medical treatment and regular follow-up with their healthcare providers [10,18]. However, the security and privacy of patient data are considered among the most reported barriers, especially when healthcare practitioners use commercial applications and services such as telemedicine systems and cloud-based telemedicine [19]. This might explain why telemedicine may not replace the traditional medicine approach, especially in the area of physical examination [10]. In addition, the lack of capacity, skill, transparency, and patient education warrant further consideration when implementing telemedicine [9].

The COVID-19 pandemic has brought a global positive change as it could accelerate the demonstration of this tool [10]. Telemedicine has become mandatory in some developed countries to offer high-quality healthcare and ensure patient safety [20]. It offers a promising option to facilitate and improve healthcare delivery [21], even for LMICs in the Middle Eastern region. A recent study showed that teledermatology could be applied during the COVID-19 pandemic in Egypt. It was an effective remote method of treatment, which could decrease the risk of COVID-19 transmission to HCPs, patients, and workers [22]. However, knowledge about the experience of HCPs in Egypt and other Arabic countries with telemedicine is still scarce. Exploring HCPs’ experience with telemedicine technology, in terms of their view of its advantages and disadvantages, can reflect their acceptance and willingness to use this technology. Additionally, exploring the barriers that negatively affect their experience can guide the development of a strategic plan to mitigate these barriers. However, there is no culturally adapted and validated tool that can provide policymakers with basic information about HCPS’ perceptions of telemedicine services and allow the identification of barriers to the use of telemedicine in Egypt.

### Purpose

Our study aims to develop and investigate the psychometric properties of the Perceived Telemedicine Importance, Disadvantages, and Barriers (PTIDB) questionnaire in Arabic to be used for national surveys to assess the perception of HCPs (who have used or not used telemedicine) in Egypt about telemedicine and its barriers of utilization.

## 2. Development and Pre-Testing of the PTIDB Questionnaire

The study was performed following three phases [23,24].

### 2.1. Phase-I: Development of the Questionnaire

Three steps were conducted to achieve this phase.

We initially explored the literature to find the existing validated questionnaires on the perception of HCPs with telemedicine technology. This step was necessary to find related constructs and items that could contribute to developing a culturally relevant and valid questionnaire. Thus, questionnaires that focused only on HCPs were reviewed. Of the few questionnaires that were developed to assess HCPs’ satisfaction with telemedicine or telehealth [25,26,27,28,29], none have been developed or validated for HCPs in Arab countries.

Second, an initial version of the questionnaire, which involved a pool of 30 items, was generated based on the previous literature [25,26,27,28,29] and three of the authors’ (NY, M. Alb, and M. Abd) inputs. The 30 items (questions) were distributed under four domains (Factors) as follows: Domain-1 ‘‘importance of using telemedicine’’, Domain-2 ‘‘advantages of telemedicine’’, Domain-3 ‘‘disadvantages of telemedicine’’, and Domain-4 ‘‘barriers to utilizing telemedicine’’, alongside one item for assessing preference for utilizing telemedicine over the traditional way.

Third, an expert panel with telemedicine and questionnaire structure background evaluated the questionnaire for face and content validity. They assessed the items and domains and made suggestions regarding the (i) adequacy and relevancy of the items that were selected, (ii) adequacy and relevancy of domains, and (iii) correct language for better understanding. After four rounds of evaluation and discussion, five items were removed because they were considered redundant or unnecessary, a few were corrected to be grammatically clearer, and a few were modified to be more understandable.

Finally, the second version included a total of 25 items: 24 items reflected the previously listed domains, along with one item assessing preference. It was reevaluated by the expert panel before the preliminary phases and they decided that the items were clear and no further corrections were needed.

The Arabic version was translated into English using the forward and back translation approach (results not presented).

### 2.2. Phase-2: Preliminary Testing of the Questionnaire (Cognitive Interview)

For pre-testing, the penultimate version of the PTIDB questionnaire was built up and investigated by a pilot sample of HCPs (n = 10) who were invited to participate via WhatsApp. They were asked to fill out the electronic questionnaire to evaluate its clarity and understandability and to report any technical issues with filling it in and its completion time. All the participants agreed that the questionnaire was clear and understandable, and the completion time was approximately 10 min. Therefore, no further adjustments were made.

### 2.3. Phase-3: Investigating the Validity and Reliability of the Questionnaire Using a Large Survey

A cross-sectional survey was conducted to administer the PTIDB questionnaire to a large sample of HCPs to test its validity and reliability.

## 3. Materials and Methods

### 3.1. Design and Setting

An online cross-sectional survey was employed to achieve the aim of this study. The research targeted HCPs and clerks that were working in healthcare facilities such as university hospitals, private hospitals, and medical centers in Egypt.

### 3.2. Population, Criteria, and Sample Size

**Figure 1:**
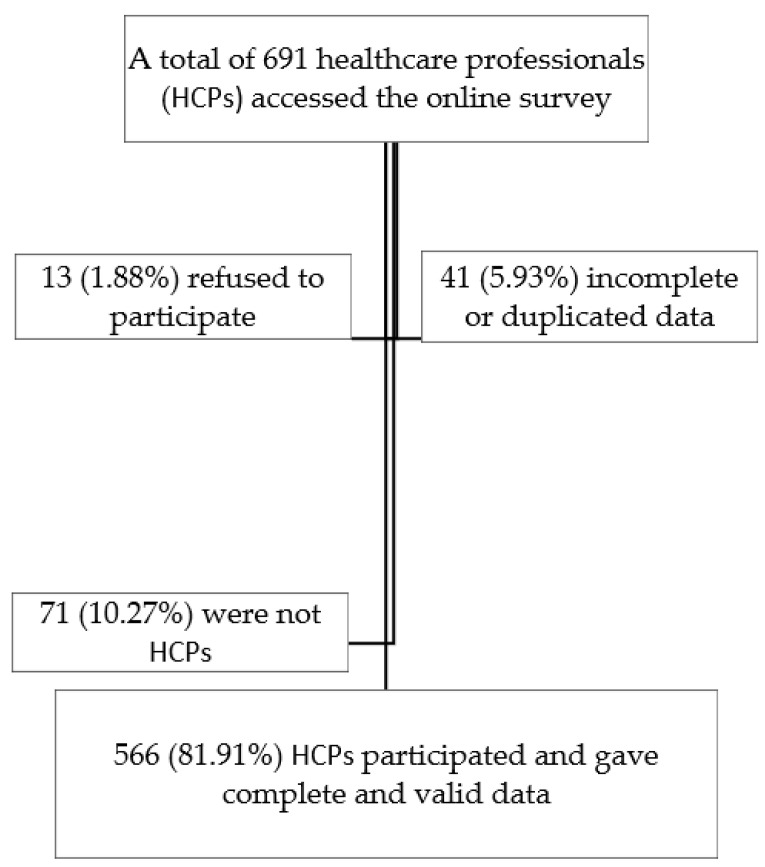
Sample flowchart.

A convenience snowball sample of HCPs was invited to participate in this study. The participants were required to meet the following criteria to participate in this study: ≥18 years of age, residence in Egypt during the period of data collection, and consent before filling in the questionnaire. The sample size was calculated based on statistical tests and the number of questionnaire items (n = 25) [30]. A maximum ratio of 20 cases per variable was applied to determine the appropriate sample for conducting the principal component analysis (PCA) [31]. The formula that was introduced in [32] was used to estimate the adequacy of the sample size for Cronbach’s alpha test to achieve a power of 80% and an alpha value of 0.05 for items that remained after the factorial structure test [33]. A total of 691 HCPs accessed the online survey over two months (June to July 2020) (Figure 1). Therefore, the sample size was sufficiently large for the used statistical tests.

### 3.3. Instruments

An anonymous electronic survey was used to collect the data. The questionnaire contained the following three sections.

Demographic and telemedicine experience sheet: This contained 10 items that collected personal data (seven items) and previous encounters with telemedicine (three items).

The PTIDB questionnaire: This was constructed of 24 items under four domains, alongside one item assessing preference. Domain-1 (five items) assessed the perception of the importance of using telemedicine. Domain-2 (six items) assessed the perception of the advantages of using telemedicine. Domain-3 (seven items) assessed the perceived disadvantages of telemedicine technology. Domain-4 (six items) assessed the perceived barriers to utilizing telemedicine. The PTIDB is a self-reporting questionnaire in which the participants can give their responses to all items (i.e., Domains 1 to 4) using a 5-point Likert scale (1 = strongly disagree, 2 = disagree, 3 = neutral, 4 = agree, and 5 = strongly agree). The total score for each domain (i.e., Domains 1–4) was calculated by summing the responses, with a higher score indicating a higher perception of importance, greater perception of advantages, greater disadvantages, and lower barriers.

A Visual Analogue Scale (one item) was used to assess the participants’ preference for utilizing telemedicine over the traditional method. The scale ranged from 0 to 10, where 0 means “I do not like it at all” and 10 means “I like it to a great extent”.

The PTIDB questionnaire has a readability level of 12th grade (https://readabilityformulas.com/freetests/six-readability-formulas.php, accessed on 26 September 2022 ).

### 3.4. Statistical Analysis

The statistical package of social science SPSS (edition 25, Chicago, IL, USA) and SPSS AMOS edition 24 were used to run all the analyses. The mean and standard deviation (SD) were calculated for continuous data, whereas categorical data were summarized using counts and percentages. Descriptive statistics were used to depict the participants’ characteristics.

The psychometric properties of the PTIDB were investigated using the following tests: (1) An exploratory factor analysis (EFA) using principal component analysis (PCA) was used to explore the factorial structure of the questionnaire using oblique direct Oblimin rotation with Kaiser normalization as the rotation method. Direct Oblimin was the rotation selected for the analysis, as the domains of the PTIDB questionnaire are likely to be associated. Sorted factor loadings, Eigenvalues, and scree plots were used to identify the number of factors and their item loading. Items with a factor loading of no less than 0.40 were considered to have acceptable factor power [34]. (2) The confirmatory factor analysis (CFA) was carried out to assess how well the EFA-identified factor structure fitted the observed data. Structural equation modelling (SEM) was used to evaluate the convergent and discriminant validity of the constructs and model fit metrics. The comparative fit index (CFI) > 0.9 and root mean square error of approximation (RMSEA) = 0.08 were the utilized criteria. (3) Discriminative (known group) validity was determined using an independent *t*-test or analysis of variance (ANOVA) according to the number of subgroups to confirm whether PTIDB could discriminate between participants according to the year of experience and speciality. A chi-square test was used to analyze non-numerical variables. (4) Item analysis was performed using the corrected item-total correlation to evaluate the convergent validity. Items must meet these criteria to be retained or deleted: (i) the item was loaded at 0.4 or more on a factor, (ii) the item did not load at more than 0.5 on two factors, and (iii) the item to the total mean score correlation was ≥ 0.5 [35]. (5) The internal reliability of each domain of the PTIDB was assessed using Cronbach’s alpha to determine its internal consistency, with an alpha value ≥ 0.70 considered acceptable [31]. A *p*-value of less than 0.05 was set as the indicator of a significant relationship.

### 3.5. Ethical Considerations

The institutional review board approved this study prior to data collection (approval reference 00208/2020). A cover page explaining the nature of the study was added to the front of the electronic survey form to help the participants familiarize themselves with the study’s purpose before providing their consent. The participants were informed that all the data that were collected would be confidential and that no specific personal data were collected during this study. The participants were also informed of their right to withdraw from the study without any consequences. Informed consent was obtained from each participant prior to administering the questionnaire. The data that were collected in this study were securely protected using a password-secured laptop computer that was accessible only to the researchers. This study was performed per the principles of the Declaration of Helsinki.

## 4. Results

### 4.1. Participants’ Sociodemographic Characteristics

More than half (54.42%) of the participants were male that were aged 36.18 years (±8.93). Of these, 23.50% lived in Cairo, the capital city of Egypt, about two-thirds were physicians (65.5%), and 45.8% were working at academic institutions or university hospitals (Table 1).

### 4.2. Utilization of Telemedicine among the Study Participants 

**Figure 2:**
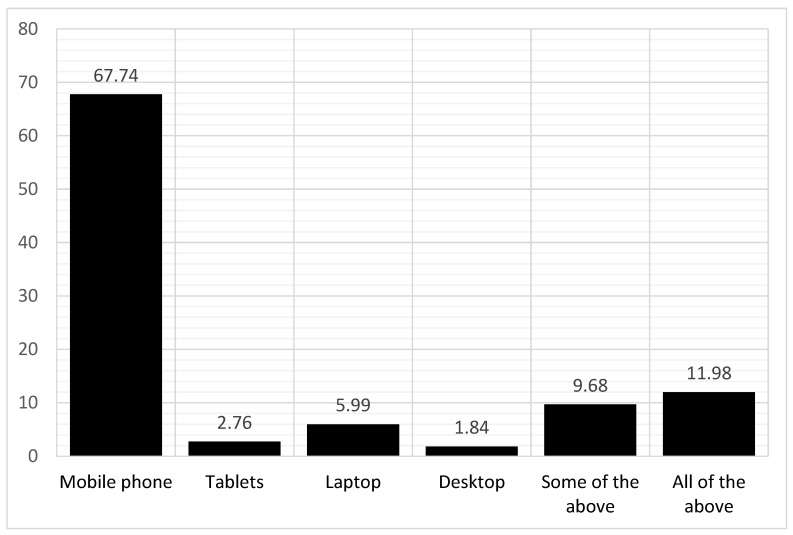
Tools that were used for telemedicine.

Of the 566 participants, 217 (38.3 %) used telemedicine. Figure 2 shows that mobile phones were the most used devices in telemedicine (67.74%), followed by laptops (5.99%), and tablets (2.76%). Of note, the least used tool was a desktop computer (1.84%). Approximately 10% (9.68%) used some of the aforementioned devices, whereas 11.98% used all of these devices. Using a preference scale ranging from zero to 10, 6.9% of the participants did not prefer the utilization of telemedicine overall, whereas the rest preferred it.

### 4.3. Association between Sociodemographic Variables and Using Telemedicine

Table 2 presents the associations between the sociodemographic variables and telemedicine use. The participants who used telemedicine were statistically older than those who did not use it (t = −2.01, *p* = 0.009). There was no statistically significant difference between males (40.3%) and females (36.0%) in the utilization of telemedicine (χ2 = 1.05, *p* = 0.305). Participants who are living in Cairo used telemedicine more frequently than Participants who are living in other governorates (43.6% vs. 36.7%). However, this difference was not statistically significant (χ2 = 2.04, *p* = 0.153). The profession of HCP was significantly associated with the usage of telemedicine; nurses were the least likely to use telemedicine (20.6%) compared to physicians and technicians (45.0% for both), and this difference was statistically significant (χ^2^ = 28.26, *p* = 0 < 0.001). The place of work was not associated with the use of telemedicine (*p* > 0.05). Finally, participants who used telemedicine reported a longer duration of work experience; however, this finding was not statistically significant (t = 171, *p* = 0.087)**.**

### 4.4. Exploratory Factor Analysis (EFA)

The EFA was conducted in the form of principal component analysis (PCA) with an oblique direct Oblimin rotation to assess the factor correlation matrix of the 24 items and to check the discriminant validity. The structural matrix revealed that 19 of the items loaded highly on the relevant proposed factors. There were five items that were deleted since their removal resulted in greater internal consistency of the questionnaire.

The rest of the ‘‘Advantages items’’ and the items of ‘‘Importance domains’’ loaded on one factor. The initial Eigenvalues showed that 19 items of the questionnaire explained 56.0% of the variance in the three extracted factors. Table 3 shows the factor loadings for items (n = 19 items) of the questionnaire. For “Factor 1”, eight items were loaded on one factor with loading ranging from 0.61 to 0.78. For “Factor 2,” seven items were loaded on one factor with factor loading ranging from 0.60 to 0.79. For “Factor 3,” four items were loaded on one factor, with factor loading ranging from 0.60 to 0.86.

### 4.5. Divergent Validity

There were both negative and positive correlations between these three factors. The largest negative correlation was between Factor 1 “Importance” and Factor 2 “Disadvantages’’ (r = −0.49), while the smallest negative correlation was between Factor 2 and Factor 3 ‘‘Barriers’’ (r = −0.32). Factors 1 and 3 showed a significant positive correlation (r = 0.41). No correlation coefficient was larger than 0.7 [36] Hence, the factors that were derived from EFA revealed adequate discriminant validity (Table 4).

Moreover, the correlations between the preference for utilizing telemedicine score and the three factors of the PTIDB questionnaire revealed that the preference for utilizing telemedicine was positively associated with ‘‘Importance’’ (r = 0.53, *p* < 0.001) and ‘‘Barriers’’ (r = 0.30, *p* < 0.001) and it was negatively associated with ‘‘Disadvantages’’ r = 0.50, *p* < 0.001).

### 4.6. Confirmatory Factor Analysis (CFA)

**Figure 3:**
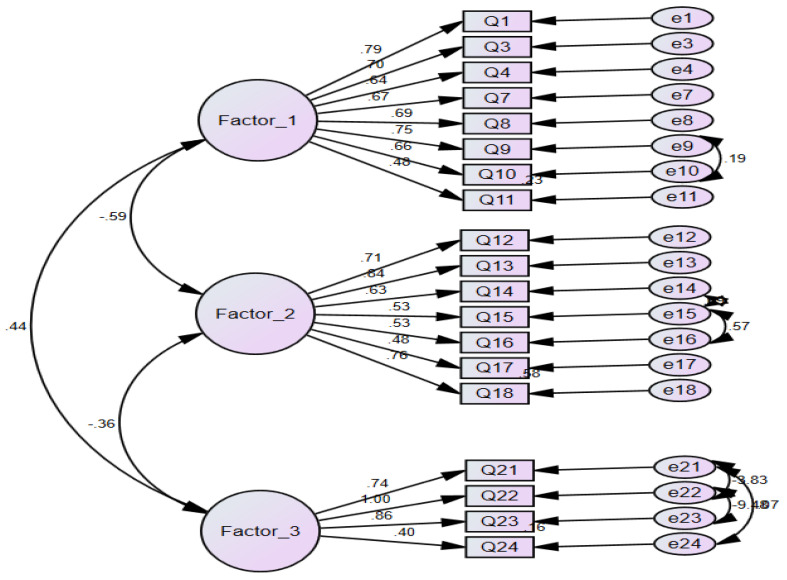
Confirmatory factor analysis of the PTIDB questionnaire.

The CFA was used to confirm the hypothesis that the questionnaire contains three factors, and to confirm that the 19 items were loaded under the relevant factor, as described in the EFA. Figure 3 shows the final CFA model using the SEM. All loadings ranged from 0.4 to 1.0, with CFI = 0.93 and RMSEA = 0.061.

### 4.7. Discriminant Validity

**Figure 4:**
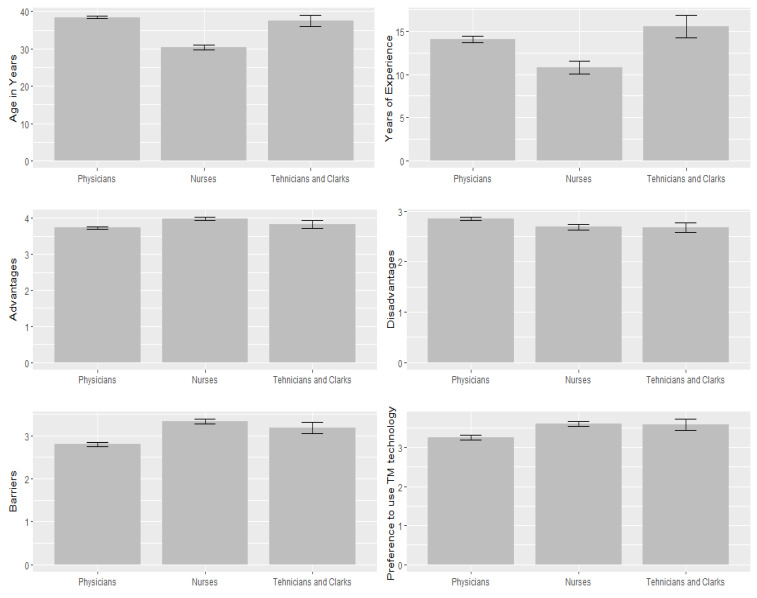
Comparing PTIDB domains according to the healthcare professional speciality.

A comparison between the study participants (i.e., physicians, nurses, and clerks) according to age, years of experience, and domains of the PTIDB questionnaire is presented in Table 5 and Figure 4. Nurses had a significantly higher level of preference for telemedicine than physicians and clerks (f = 19.452, *p* < 0.001); they also had a higher perception of telemedicine importance than physicians and clerks (f = 11.494, *p* < 0.001). In contrast, physicians had a higher perception of the disadvantages and barriers to telemedicine use than nurses and clerks (f = 8.324, & 27.191, *p*< 0.004, respectively). 

### 4.8. Reliability Analysis and Convergent Validity

The three factors had satisfactory reliability, with Cronbach’s alpha ranged from 0.79 to 0.87. All questions showed a statistically significant correlation with their related factors and all items within each factor had a significant positive correlation (Table 6). Split-half was also used to confirm the reliability of the questionnaire. The questionnaire items were randomly divided into two halves: odd questions (Q1, Q4, Q8, Q10, Q12, Q14, Q16, Q18, Q22, Q24) and even questions (Q3 Q,7, Q9, Q11, Q13, Q15, Q17, Q21, Q23). The correlation of the score of the two halves was calculated, where the score of the first half was 33.4 ± 3.4, and the second half was 29.4 ± 3.4. Finally, the calculated correlation was adjusted to estimate the reliability using the Spearman–Brown formula (r = 0.65, *p* < 0.001)

## 5. Discussion

Little is known about the perspectives of HCPs regarding telemedicine in Egypt. Therefore, researchers and policymakers in Arabic countries need a questionnaire that is easy to use, understandable, and valid. Prior to this study, there was no valid questionnaire for such assessment. This study aimed to develop and validate a new questionnaire was named the PTIDB for Arabic-speaking HCPs. The PTIDB questionnaire was designed in three phases, following which its construct validity and internal consistency were evaluated. Hence, this study is the first to design and produce a valid telemedicine questionnaire for Arabic HCP speakers in the Middle East. In this study, Arabic was used to guarantee good comprehension. The results yielded excellent construct validity and internal consistency. Factor analysis extracted three factors that suggested the multidimensionality of the PTIDB. Each factor of the PTIDB had satisfactory reliability with a Cronbach’s α that exceeded the limited criterion of 0.70 [31]. The PTIDB was developed for use in evaluating the HCPS’ perspectives of telemedicine’s importance, disadvantages, and barriers to its use.

### 5.1. Utilization of Telemedicine and Associated Factors

More than one-third of the participants used telemedicine, with mobile phones being the most used device in telemedicine. Interestingly, the least used devices were desktop computers, indicating that the infrastructure and facilities for using telemedicine in the workplace are not well established. However, the majority of the study participants preferred to utilize telemedicine. Since the capital city has more resources and the latest technology compared to other governorates, it is logical to find that participants living in Cairo use telemedicine more frequently than those living in other areas. This finding also suggests that resources, as well as training of the staff, might be necessary factors that must be taken into consideration to overcome barriers to utilizing telemedicine.

### 5.2. Construct Validity of the PTIDB

The CFA verified the EFA output and provided an initial proof of the construct validity of the PTIDB. The PTIDB seems to be a multidimensional questionnaire, giving the advantage of using each of its domains independently. However, future studies are needed to test the hypothesis of its multidimensionality.

### 5.3. Divergent Validity of the PTIDB

The findings of this study showed that there were no correlation coefficients larger than 0.7; hence, the factors derived from EFA revealed adequate discriminant validity. The largest negative correlation was found between importance and disadvantages, which revealed that the perceived importance of telemedicine utilization decreased when the perception of telemedicine disadvantages increased. The smallest negative correlation was noted between disadvantages and barriers, suggesting that the perception of barriers to telemedicine utilization increased when perceived disadvantages increased. Moreover, importance and barriers had a significant positive correlation, which demonstrating that when barriers to telemedicine decreased, the sense of importance increased. Also, our study found that the preference for using telemedicine increased when the perception of disadvantages and barriers decreased.

### 5.4. Discriminant (Known Group) Validity

Compared with physicians and clerks, nurses had a significantly higher level of preference for the use of telemedicine technology and a higher perception of telemedicine importance. However, physicians had a higher perception of the disadvantages and barriers to telemedicine use than nurses. These findings are consistent with a previous study [37] that showed that nursing staff were more likely to accept telehealth, and physicians were more likely to worry about their professional practice and how it might be underestimated [37]. Another study showed that senior physicians had a limited interest in telemedicine technology, and several of them believed that telemedicine could not improve clinical practice [38]. The suggested reason why nurses are more open to the use of telemedicine, while physicians are more resistant to it, might be related to the frequency of use. In Egypt, physicians apply telemedicine more often than nurses do. Therefore, it is logical to conclude that physicians had a higher perception of telemedicine barriers than nurses, which might be related to the limitations of resources and training. HCPs’ preference for telemedicine and barriers to its use have been acknowledged as important determinants of successful telemedicine implementation [39]. Therefore, the perception of facilitators must be taken into consideration to increase HCPs (nurses’ and physicians’) intentions to use advanced technology [39]. Thus, this finding bears direct implications on the relevance of the proposed instrument, which might suggest the known group validity of the PTIDB questionnaire. However, further studies are needed to confirm the discriminant sensitivity of the PTIDB questionnaire. Testing the factor structure across groups (i.e., nurses and doctors) in a larger prospective study is recommended to add more evidence of its validity and retest reliability according to the healthcare providers’ speciality.

### 5.5. Reliability of the PTIDB

Internal consistency was assessed using Cronbach’s alpha. Internal reliability refers to the degree of accuracy of the questionnaire and the relationship between its components [40]. The internal consistency of our study was good, with a high Cronbach’s alpha coefficient of >0.7, indicating good-to-excellent internal consistency [31].

### 5.6. Strengths, Limitations, and Conclusions

The development of the PTIDB questionnaire was the main contribution of this study, where it can be used to evaluate HCPs’ perceptions of telemedicine’s importance, disadvantages, and barriers to implementation, especially in the Middle East, where Arabic is the most commonly used language. This study revealed that the PTIDB is a robust and easy-to-use questionnaire that can be administered and completed in a short amount of time by Arabic-speaking HCPs. Policymakers and researchers need a gold standard tool to enhance their understanding of HCPs’ experiences with regard to telemedicine and barriers to implementing telemedicine in their workplace. The PTIDB can be administered before and after launching and the administration of telemedicine technology to test its effectiveness in improving the adoption of this technology and its ability to implement it.

Despite the contributions of this methodological study to existing literature, several limitations should be acknowledged. First, the generalizability of the PTIDB’s findings to Arabic-speaking HCPs in other Arab countries may be limited because the sample was obtained through a convenience sampling method and was limited to participants from Egypt. Second, this study did not apply the test-retest method to guarantee retest reliability. Therefore, additional studies are needed to establish the questionnaire’s retest-reliability and discriminative validity.

In summary, this study contributes to the literature on the existence of an Arabic telemedicine questionnaire and its primary validity and reliability are supported by our study results. Permission to use the PTIDB questionnaire must be obtained from the corresponding author (NY), and all those who will use it in future studies should cite this paper. 

## Figures and Tables

**Table 1:** Sociodemographic characteristics of the participants, (n = 566).

Variable	Category	Frequency	Percent
Age (mean ± SD)			36.18 ± 8.93
Sex	Male	308	54.42
	Female	258	45.58
Residence	Cairo/Giza	133	23.50
	Other	433	76.50
Occupation	Physicians	371	65.50
	Nurses	155	27.40
	Technician and Clarks	40	7.10
Place of work	Academic Centre/university hospital	259	45.80
	General Hospital	153	27.00
	Private hospital/clinic/centers	112	19.80
	Police hospital	5	0.90
	Other	37	6.50

**Table 2:** Association between the different sociodemographic variables and telemedicine use.

Variables		Did Not Use Telemedicine 349 (61.7)	Used Telemedicine 217(38.3)	*T*-Test/Chi-Square	*p*-Value
		N (%)			
Gender	Male	184 (59.7)	124 (40.3)	1.05	0.305
	Female	165 (64.0)	93 (36.0)		
Residence	Cairo/Giza	75 (56.40)	58 (43.60)	2.04	0.153
	Other	274 (63.3)	159 (36.7)		
Job	Physicians	204 (55.00)	167 (45.00)	28.26	<0.001
	Nurses	123 (79.4)	32 (20.6)		
	Technician and Clarks	22 (55.00)	18 (45.00)		
Place of work	Academic Centre/university hospital	156 (62.20)	103 (30.80)	3.85	0.426
	General Hospital	104 (68.00)	49 (32.00)		
	Private hospital/clinic/centres	64 (51.70)	48 (42.90)		
	Police hospital	3 (60.00)	2 (40.0)		
	Other	22 (59.50)	15 (40.50)		
Age	(mean ± Std. Deviation)	35.40 ± 8.92	7.42 ± 8.82	−2.01	0.009
Year of experience		12.79 ± 8.23	14.02 ± 8.36	−0.171	0.087

**Table 3:** Factor loading of the PTIDB questionnaire.

Questionnaire’s Items	Factor 1	Factor 2	Factor 3	Communalities
Q1	0.77	−0.03	−0.08	0.67
Q3	0.78	0.04	0.01	0.57
Q4	0.76	0.11	0.00	0.52
Q7	0.72	−0.06	0.06	0.53
Q8	0.66	−0.07	−0.07	0.52
Q9	0.58	−0.25	−0.15	0.62
Q10	0.64	−0.11	−0.05	0.51
Q11	0.61	0.06	0.04	0.34
Q12	−0.12	0.70	−0.08	0.54
Q13	−0.08	0.71	0.14	0.63
Q14	−0.08	0.68	−0.07	0.49
Q15	0.14	0.78	0.05	0.56
Q16	0.16	0.79	0.06	0.56
Q17	−0.11	0.60	−0.18	0.39
Q18	−0.03	0.69	0.16	0.58
Q21	−0.08	0.02	−0.86	0.70
Q22	0.22	−0.11	−0.60	0.55
Q23	−0.06	−0.01	−0.83	0.67
Q24	0.06	0.06	−0.64	0.42

**Table 4:** Divergent validity of the PTIDB questionnaire.

Factors	Correlations Test	Factor-1	Factor-2	Factor-3
Importance of TM	r (*p*-value)	1	−0.49 (<0.001)	0.41(<0.001)
Disadvantages of TM			1	−0.32(<0.001)
Barriers to TM utilization				**1**

Correlation is significant at the 0.05 level (2-tailed). TM: telemedicine.

**Table 5:** Comparison of PTIDB domains according to the healthcare professional speciality (n = 566).

Variables		N	Mean	Std. Deviation	F	Sig.
Age	Physicians	371	38.46	7.70	43.02	<0.001
	Nurses	155	30.39	8.97		
	Technician and Clarks	40	37.50	9.44		
Year of experience	Physicians	371	14.05	7.59	2.77	0.096
	Nurses	155	10.80	9.37		
	Technician and Clarks	40	15.53	8.28		
Importance	Physicians	371	3.76	0.63	11.49	<0.001
	Nurses	155	4.00	0.51		
	Technician and Clarks	40	3.89	0.63		
Disadvantages	Physicians	371	2.94	0.68	8.32	0.004
	Nurses	155	2.74	0.70		
	Technician and Clarks	40	2.76	0.64		
Barriers	Physicians	371	2.84	0.79	27.19	<0.001
	Nurses	155	3.29	0.72		
	Technician and Clarks	40	3.16	0.87		
Preference to use TM technology	Physicians	371	4.41	3.02	19.45	<0.001
	Nurses	155	5.83	3.01		
	Technician and Clarks	40	5.50	3.26		

**Table 6:** Descriptive statistics, reliability, and convergent validity of the PTIDB questionnaire.

Factors/Items	Mean ± SD	Item to Mean Score Correlation		Cronbach’s Alpha	
		r	*p*		
Importance	3.84 ± 0.61	-	-	-	0.87
Q1	3.72 ± 0.82	0.80	0.001	0.74	
Q3	3.91 ± 0.85	0.74	0.001	0.65	
Q4	3.99 ± 0.74	0.69	0.001	0.59	
Q7	4.10 ± 0.74	0.70	0.001	0.62	
Q8	3.88 ± 0.84	0.73	0.001	0.64	
Q9	3.37 ± 1.05	0.79	0.001	0.68	
Q10	3.70 ± 0.90	0.74	0.001	0.61	
Q11	4.04 ± 0.83	0.60	0.001		
Disadvantages	2.79 ± 0.69	-	-	-	0.82
Q12	2.83 ± 0.97	0.74	0.001	0.61	
Q13	3.23 ± 1.03	0.79	0.001	0.62	
Q14	2.58 ± 0.89	0.71	0.001	0.60	
Q15	2.75 ± 0.98	0.72	0.001	0.61	
Q16	2.99 ± 0.99	0.72	0.001	0.59	
Q17	2.38 ± 0.84	0.63	0.001	0.49	
Q18	3.40 ± 1.02	0.60	0.001		
Barriers	2.99 ± 0.81	-	-	-	0.79
Q21	2.96 ± 1.14	0.83	0.001	0.63	
Q22	3.29 ± 0.96	0.73	0.001	0.54	
Q23	2.61 ± 1.11	0.81	0.001	0.62	
Q24	3.10 ± 1.01	0.69	0.001	0.55	

## Data Availability

Data are available and will be provided upon request.

## References

[1] Huang C., Wang Y., Li X., Ren L., Zhao J., Hu Y., Zhang L., Fan G., Xu J., Gu X. (2020). Clinical features of patients infected with 2019 novel coronavirus in Wuhan, China. Lancet.

[2] World Health Organization (2020). WHO Director-General’s Opening Remarks at the Media Briefing on COVID-19–11 March 2020. https://www.who.int/director-general/speeches/detail/who-director-general-s-opening-remarks-at-the-media-briefing-on-covid-19---11-march-2020.

[3] World Health Organization (2022). WHO Coronavirus (COVID-19) Dashboard | WHO Coronavirus (COVID-19) Dashboard With Vaccination Data. https://covid19.who.int.

[4] El Kassas M., Asem N., Abdelazeem A., Madkour A., Sayed H., Tawheed A., Al Shafie A., Gamal M., Elsayed H., Badr M. (2020). Clinical features and laboratory characteristics of patients hospitalized with COVID-19: Single centre report from Egypt. J. Infect. Dev. Ctries.

[5] World Health Organization WHO Health Emergency Dashboard (2022). WHO (COVID-19) Homepage. Egypt Situation. https://covid19.who.int/region/emro/country/eg.

[6] World Health Organization (2022). Egypt WHO Coronavirus Disease (COVID-19) Dashboard with Vaccination Data. https://covid19.who.int/?mapFilter=vaccinations.

[7] Medhat M.A., El Kassas M. (2020). COVID-19 in Egypt: Uncovered figures or a different situation?. J. Glob. Health.

[8] Zhao Z., Li X., Liu F., Zhu G., Ma C., Wang L. (2020). Prediction of the COVID-19 spread in African countries and implications for prevention and control: A case study in South Africa, Egypt, Algeria, Nigeria, Senegal and Kenya. Sci. Total Environ..

[9] Mahmoud K., Jaramillo C., Barteit S. (2022). Telemedicine in low-and middle-income countries during the COVID-19 pandemic: A scoping review. Front. Public Health.

[10] Perrone G., Zerbo S., Bilotta C., Malta G., Argo A. (2020). Telemedicine during Covid-19 pandemic: Advantage or critical issue?. Med.-Leg. J..

[11] Doraiswamy S., Abraham A., Mamtani R., Cheema S. (2020). Use of telehealth during the COVID-19 pandemic: Scoping review. J. Med. Internet Res..

[12] Hong Z., Li N., Li D., Li J., Li B., Xiong W., Lu L., Li W., Zhou D. (2020). Telemedicine during the COVID19 pandemic: Experiences from western China. J. Med. Internet Res..

[13] (1997). WHO Group Consultation on Health Telematics (1997: Geneva, Switzerland). (1998). A Health Telematics Policy in Support of WHO’s Health-for-All Strategy for Global Health Development: Report of the WHO Group Consultation on Health Telematics, 11–16 December, Geneva. World Health Organization. https://apps.who.int/iris/handle/10665/63857.

[14] World Health Organization Fifty-eighth World Health Assembly, Geneva: Resolutions and decisions, annex. Proceedings of the Fifty-Eighth World Health Assembly.

[15] Craig J., Patterson V. (2005). Introduction to the practice of telemedicine. J. Telemed. Telecare.

[16] Vidal-Alaball J., Garcia Domingo J.L., Garcia Cuyàs F., Mendioroz Peña J., Flores Mateo G., Deniel Rosanas J., Sauch Valmaña G. (2018). A cost savings analysis of asynchronous teledermatology compared to face-to-face dermatology in Catalonia. BMC Health Serv. Res..

[17] Langabeer J.R., Champagne-Langabeer T., Alqusairi D., Kim J., Jackson A., Persse D., Gonzalez M. (2017). Cost–benefit analysis of telehealth in pre-hospital care. J. Telemed. Telecare.

[18] Sayani S., Muzammil M., Saleh K., Muqeet A., Zaidi F., Shaikh T. (2019). Addressing cost and time barriers in chronic disease management through telemedicine: An exploratory research in select low-and middle-income countries. Ther. Adv. Chronic Dis..

[19] Jin Z., Chen Y. (2015). Telemedicine in the cloud era: Prospects and challenges. IEEE Pervasive Comput..

[20] Amudha R., Nalini R., Alamelu R., Badrinath V., Sharma M.N. (2017). Telehealth and Telenursing-Progression in healthcare practice. Res. J. Pharm. Technol..

[21] Stroetmann K.A., Kubitschke L., Robinson S., Stroetmann V., Cullen K., McDaid D. (2010). How Can Telehealth Help in the Provision of Integrated Care?.

[22] Mostafa P.I.N., Hegazy A.A. (2020). Dermatological consultations in the COVID-19 era: Is teledermatology the key to social distancing? An Egyptian experience. J. Dermatolog. Treat..

[23] Boateng G.O., Neilands T.B., Frongillo E.A., Melgar-Quiñonez H.R., Young S.L. (2018). Best practices for developing and validating scales for health, social, and behavioral research: A primer. Front. Public Health.

[24] Boparai J.K., Singh S., Kathuria P. (2018). How to design and validate a questionnaire: A guide. Curr. Clin. Pharmacol..

[25] Parmanto B., Lewis A.N., Graham K.M., Bertolet M.H. (2016). Development of the telehealth usability questionnaire (TUQ). Int. J. Telerehabil..

[26] Lund A.M. (2001). Measuring usability with the USE questionnaire. Usability Interface.

[27] Hirani S.P., Rixon L., Beynon M., Cartwright M., Cleanthous S., Selva A., Sanders C., Newman S.P. (2017). Quantifying beliefs regarding telehealth: Development of the whole systems demonstrator service user technology acceptability questionnaire. J. Telemed. Telecare.

[28] Davis F.D. (1989). Perceived usefulness, perceived ease of use, and user acceptance of information technology. MIS Q..

[29] Ayatollahi H., Sarabi F.Z.P., Langarizadeh M. (2015). Clinicians’ knowledge and perception of telemedicine technology. Perspect. Health Inf. Manag..

[30] Kyriazos T.A. (2018). Applied psychometrics: Sample size and sample power considerations in factor analysis (EFA, CFA) and SEM in general. J. Psychol..

[31] Nunnally J.C. (1978). Psychometric Theory.

[32] Bonett D.G. (2002). Sample size requirements for testing and estimating coefficient alpha. J. Educ. Behav. Stat..

[33] Bujang M.A., Omar E.D., Baharum N.A. (2018). A review on sample size determination for Cronbach’s alpha test: A simple guide for researchers. Malays. J. Med. Sci..

[34] Hair J.F., Anderson R.E., Babin B.J., Black W.C. (2009). Multivariate Data Analysis: A Global Perspective.

[35] Gulledge C.M., Smith D.G., Ziedas A., Muh S.J., Moutzouros V., Makhni E.C. (2019). Floor and ceiling effects, time to completion, and question burden of PROMIS CAT domains among shoulder and knee patients undergoing nonoperative and operative treatment. JBJS Open Access.

[36] Pillai N.V., Rjumohan A. (2020). Reliability, Validity and Uni-Dimensionality: A Primer. https://mpra.ub.uni-muenchen.de/id/eprint/101714.

[37] MacNeill V., Sanders C., Fitzpatrick R., Hendy J., Barlow J., Knapp M., Rogers A., Bardsley M., Newman S.P. (2014). Experiences of front-line health professionals in the delivery of telehealth: A qualitative study. Br. J. Gen. Pract..

[38] Gaggioli A., Di Carlo S., Mantovani F., Castelnuovo G., Riva G. (2005). A telemedicine survey among Milan doctors. J. Telemed. Telecare.

[39] Gagnon M.P., Orruño E., Asua J., Abdeljelil A.B., Emparanza J. (2012). Using a modified technology acceptance model to evaluate healthcare professionals’ adoption of a new telemonitoring system. Telemed. e-Health.

[40] DeVellis R.F., Thorpe C.T. (2021). Scale Development: Theory and Applications.

